# New Variant of MELAS Syndrome With Executive Dysfunction, Heteroplasmic Point Mutation in the *MT-ND4* Gene (m.12015T>C; p.Leu419Pro) and Comorbid Polyglandular Autoimmune Syndrome Type 2

**DOI:** 10.3389/fimmu.2019.00412

**Published:** 2019-03-21

**Authors:** Dominique Endres, Patrick Süß, Simon J. Maier, Evelyn Friedel, Kathrin Nickel, Christiane Ziegler, Bernd L. Fiebich, Franz X. Glocker, Friedrich Stock, Karl Egger, Thomas Lange, Michael Dacko, Nils Venhoff, Daniel Erny, Soroush Doostkam, Katalin Komlosi, Katharina Domschke, Ludger Tebartz van Elst

**Affiliations:** ^1^Section for Experimental Neuropsychiatry, Department of Psychiatry and Psychotherapy, Medical Center-University of Freiburg, Faculty of Medicine, University of Freiburg, Freiburg, Germany; ^2^Department of Psychiatry and Psychotherapy, Medical Center-University of Freiburg, Faculty of Medicine, University of Freiburg, Freiburg, Germany; ^3^Department of Neurology, Medical Center-University of Freiburg, Faculty of Medicine, University of Freiburg, Freiburg, Germany; ^4^Institute of Human Genetics, University Hospital Muenster, Muenster, Germany; ^5^Department of Neuroradiology, Medical Center-University of Freiburg, Faculty of Medicine, University of Freiburg, Freiburg, Germany; ^6^Department of Radiology, Medical Physics, Medical Center-University of Freiburg, Faculty of Medicine, University of Freiburg, Freiburg, Germany; ^7^Department of Rheumatology and Clinical Immunology, Medical Center-University of Freiburg, Faculty of Medicine, University of Freiburg, Freiburg, Germany; ^8^Institute of Neuropathology, Medical Center-University of Freiburg, Faculty of Medicine, University of Freiburg, Freiburg, Germany; ^9^Berta-Ottenstein-Programme, Faculty of Medicine, University of Freiburg, Freiburg, Germany; ^10^Institute of Human Genetics, Medical Center-University of Freiburg, Faculty of Medicine, University of Freiburg, Freiburg, Germany

**Keywords:** MELAS, mitochondrial disease, polyglandular autoimmune syndrome, *ND4* gene, dysexecutive syndrome

## Abstract

**Background:** Mitochondrial diseases are caused by dysfunctions in mitochondrial metabolic pathways. MELAS syndrome is one of the most frequent mitochondrial disorders; it is characterized by encephalopathy, myopathy, lactic acidosis, and stroke-like episodes. Typically, it is associated with a point mutation with an adenine-to-guanine transition at position 3243 of the mitochondrial DNA (mtDNA; m.3243A>G) in the mitochondrially encoded tRNA leucine 1 *(MT-TL1)* gene. Other point mutations are possible and the association with polyglandular autoimmune syndrome type 2 has not yet been described.

**Case presentation:** We present the case of a 25-year-old female patient with dysexecutive syndrome, muscular fatigue, and continuous headache. Half a year ago, she fought an infection-triggered Addison crisis. As the disease progressed, she had two epileptic seizures and stroke-like episodes with hemiparesis on the right side. Cerebral magnetic resonance imaging showed a substance defect of the parieto-occipital left side exceeding the vascular territories with a lactate peak. The lactate ischemia test was clearly positive, and a muscle biopsy showed single cytochrome c oxidase-negative muscle fibers. Genetic testing of blood mtDNA revealed a heteroplasmic base exchange mutation in the mitochondrially encoded NADH:ubiquinone oxidoreductase core subunit 4 *(MT-ND4)* gene (m.12015T>C; p.Leu419Pro; heteroplasmy level in blood 12%, in muscle tissue: 15%). The patient suffered from comorbid autoimmune polyglandular syndrome type 2 with Hashimoto's thyroiditis, Addison's disease, and autoimmune gastritis. In addition, we found increased anti-glutamic acid decarboxylase 65, anti-partial cell, anti-intrinsic factor, and anti-nuclear antibodies.

**Conclusion:** We present an atypical case of MELAS syndrome with predominant symptoms of a dysexecutive syndrome, two stroke-like episodes, and fast-onset fatigue. The symptoms were associated with a not yet described base and aminoacid exchange mutation in the *MT-ND4* gene (m.12015T>C to p.Leu419Pro). The resulting changed protein complex in our patient is part of the respiratory chain multicomplex I and might be the reason for the mitochondriopathy. However, different simulations for pathogenetic relevance are contradictory and rather speak for a benign variant. To our knowledge this case report is the first reporting MELAS syndrome with comorbid polyglandular autoimmune syndrome type 2. Screening for autoimmune alterations in those patients is important to prevent damage to end organs.

## Background

Mitochondrial diseases are caused by malfunctions surrounding the mitochondrial metabolic pathways. They are clinically, genetically, and biochemically heterogeneous, can occur at any age, often involve the central nervous system (CNS), and are usually associated with multiorgan involvement (1, 2). Mitochondria produce energy for the cell in form of adenosine triphosphate (ATP) generated by oxidative phosphorylation within the respiratory chain. The respiratory chain is composed of five enzymatic multiprotein complexes (I, II, III, IV, and V). Four of these complexes contain both proteins encoded by nuclear DNA (nDNA) and proteins encoded by mitochondrial DNA (mtDNA). Therefore, mutations in nuclear and mitochondrial DNA may affect the respiratory chain function resulting in impaired ATP synthesis and cellular dysfunction or death (3). The mtDNA mutations can be divided into structural rearrangements (e.g., deletions), quantitative disturbances of mtDNA also being called mtDNA depletion (reduction of the mtDNA copy number), and point mutations (base exchange mutations) (1). Some known inherited mitochondrial disorders are MELAS (Mitochondrial Encephalomyopathy, Lactic Acidosis, and Stroke-Like Episodes), LHON (Leber hereditary optic neuropathy), or MERRF (myoclonic epilepsy and ragged red fibers) (1, 2). Because of the mixed genetics and the broad spectrum of biochemical functions of mitochondria the clinical manifestations of mitochondrial disorders are very heterogeneous. Typically, post-mitotic long-living cells like neurons, muscle cells or retinal cells are predominantly affected by these diseases. MELAS, one of the most common mitochondrial disorders, is characterized mainly by symptoms of myopathy, encephalopathy, lactic acidosis, stroke-like episodes, and other organ-related complications such dementia, epilepsy, headache, hearing impairment, short stature, or type 1 diabetes mellitus (3, 4).

Autoimmune polyendocrine syndromes (APS) are a heterogeneous family of autoimmune diseases. The two major types, APS-1 and APS-2 are both associated with adrenal cortex insufficiency (Addison's disease). While APS type 2 is most frequently associated with diabetes mellitus and/or autoimmune thyroiditis, APS type 1 is often characterized by the coexistence of hypoparathyroidism and mucocutaneous candidiasis, a condition which is also called autoimmune polyendocrinopathy candidiasis ectodermal dystrophy (APECED). Both types can be associated with other autoimmune diseases (5, 6).

### Mitochondrial Diseases and Autoimmunity

The most prevalent immunological alteration in mitochondrial disease is type 1 diabetes mellitus, which is associated with the point mutation m.3243A > G and is frequently found in patients with mitochondrial disease (7, 8). Autoimmune polyglandular syndrome type 2 (with Addison's disease, autoimmune thyroiditis, diabetes mellitus, and primary ovarian failure) was reported once in a patient with mitochondrial disease, more specifically in a female patient with KSS and a consistent 2,532-bp deletion of her mtDNA, and—beyond that—with a heteroplasmic m.3243A > G mutation (9)—but not in patients with MELAS syndrome.

## Case Presentation

We present the case of a 25-year-old female German patient with dysexecutive syndrome (affective instability, sensory overload, concentration, and impulse control problems), rapid-onset physical fatigue, and continuous headache.

### History of Illness

The patient's history was negative for *in-utero* or birth complications, inflammatory brain diseases, or febrile convulsions. She reported cognitive deficits (memory difficulties and needing more time to learn than her siblings) and impulsive behaviors (especially when things change) since her first decade of life. Both symptoms were mild and did not meet the diagnostic criteria for attention-deficit/hyperactivity disorder or autism. The subject did not encounter tics, or extraordinary personality traits, though she had known a physical lack of energy for as long as she could remember. She was easily exhausted in sports at school and could not keep up with her family on hikes. She had to go to bed early, upon which she fell asleep swiftly. Since she was 6 years old, she experienced repeated syncopations which based on history probably represented vasovagal pathomechanisms. There was no history of increased frequency of infections, especially no signs of mucocutaneous candidiasis. Dental history was unremarkable.

**Figure 1:**
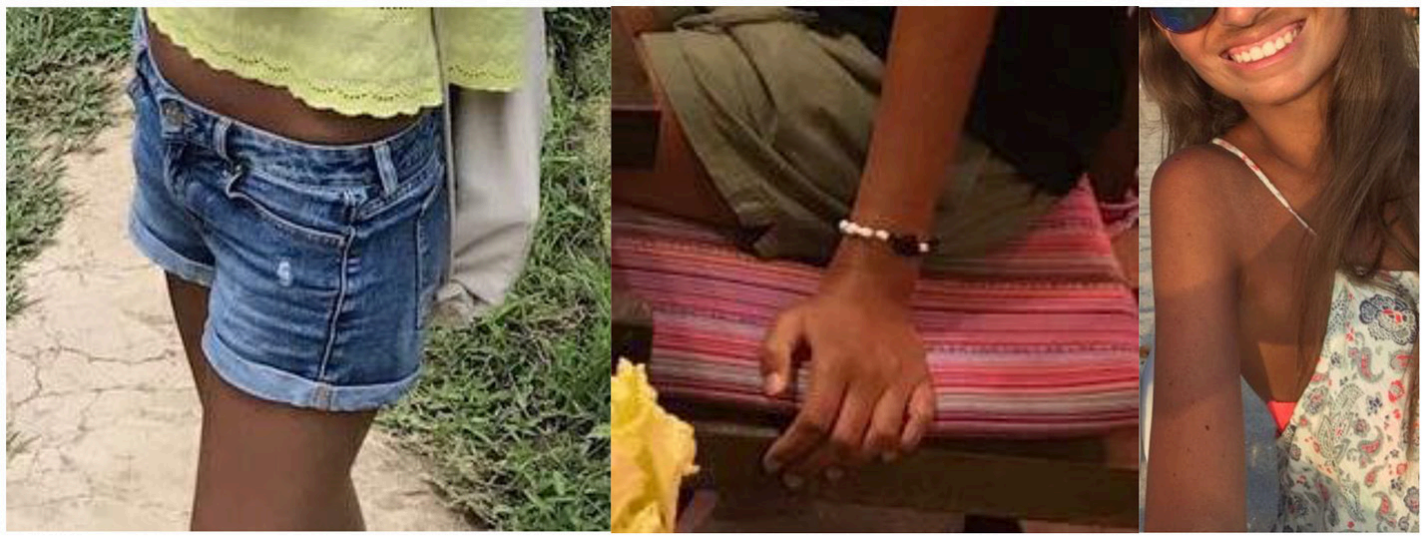
Hyperpigmentation and skinny figure due to Addison's disease.

**Figure 2:**
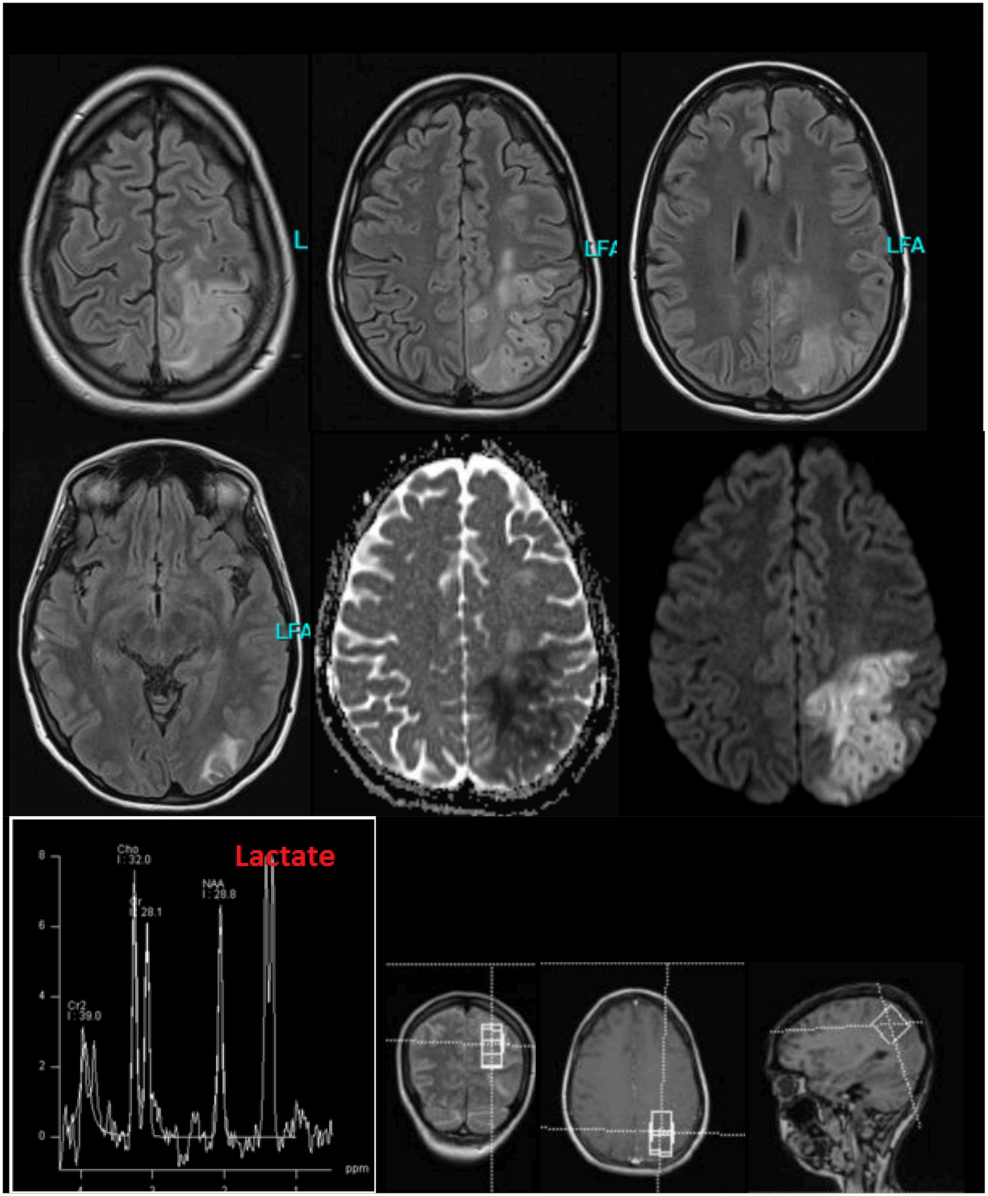
Cerebral magnetic resonance imaging (MRI) findings during the stay in the intensive care unit because of stroke like episodes. The MRI showed a subacute left parieto-occipital cortical/subcortical lesion involving the precentral gyrus with T2-weighted FLAIR signal increase and diffusion restriction without respecting vascular territories and the multiple right-hemispheric chronic posttraumatic lesions. The former subdural hemorrhage showed complete resolution. In the lesion, an increased lactate peak was registered (uncorrected data).

When she was seventeen, she fell off her bike in an unclear way and suffered brain trauma with cerebral hemorrhage. The cerebral magnetic resonance imaging (MRI) showed left hemispheric subdural hemorrhage and “contre-coup” right fronto-opercular as well as multiple right temporal cerebral contusions. Neither a subsequent change of personality nor other neurocognitive or psychomotor symptoms were observed by the patient or her family. Five years ago, she noticed hyperpigmentation (Figure 1). Four years ago, she developed early morning nausea that occurred almost on a daily base. She had no appetite and could eat very little. At age 25 (half a year before presentation in our clinic), she developed an infection-triggered Addison crisis with hypotension, diarrhea/vomiting, hypoglycemia, vigilance reduction, and metabolic acidosis. The diagnosis of Addison's disease was suspected due to the Addison crisis, anti-adrenal cortex autoantibodies (titer of 1:100; reference < 1:10) and indicative laboratory values (ACTH increased, 71.4 pg/ml; reference: 5–50 pg/ml). During her inpatient stay, the patient already noticed a self-limiting, discrete hemiparesis on the right side. Treatment was started with a glucocorticoid (hydrocortisone) and a mineralocorticoid (fludrocortisone). Later, the patient developed two grand mal seizures, for which she was admitted to an intensive care unit. An anti-epileptic treatment with levetiracetam was initiated. While being on intensive care treatment, she developed progressive disorientation, reduced vigilance, headache, coordination disturbances and, again, hemiparesis on the right side. The MRI showed a subacute left parieto-occipital cortical/subcortical lesion involving the precentral gyrus with T2-weighted FLAIR signal increase and diffusion restriction without respecting vascular territories and the multiple right-hemispheric chronic posttraumatic lesions. There was a clear lactate peak in the lesion (Figure 2). The EEG showed an intermittent slowing temporoparietal on both sides (stronger on the left) and singular spike-wave complexes temporal (once temporoparietal) on the left. During her subsequent stay in a neurological rehabilitation clinic, her neurological deficits receded almost completely. On occasion her right hand may feel clumsier than her left, but this occurs rarely. Following the grand mal seizures symptoms of sensory overload and affective instability evolved and persisted ever since. She has not experienced any further early morning nausea following treatment with hydro- and fludrocortisone. Several attempts to reduce levetiracetam led to an exacerbation of cognitive deficits.

### Somatic and Family History

The patient suffered from Hashimoto's thyroiditis for years (substituted with L-thyroxine) and, as already mentioned, Addison's disease. When being diagnosed for the Addison crisis normochromic, normocytic anemia was recognized (hemoglobin: 10.4 g/dl; reference 12–16 g/dl). Two years prior to present admission a gastroscopy was done revealing mild to moderate antrum gastritis suspicious of chronic toxic (type C) gastritis. Her mother, sister, and maternal grandfather all suffered from type 1 diabetes mellitus, while another sister is healthy.

### Genetic Investigations

Analysis of the complete mitochondrial genome from peripheral blood leukocytes showed a heteroplasmic base exchange mutation in the mitochondrially encoded NADH:ubiquinone oxidoreductase core subunit 4 *(MT-ND4)* gene (m.12015T>C; p.Leu419Pro). Heteroplasmy level for the mutation in blood showed 12%, in muscle tissue 15%. This point mutation was not detected in the blood of the patient's mother, no other tissues were analyzed in the mother. No other pathogenic or likely pathogenic alteration was detected in the mtDNA of the patient.

In MITOMAP, the human mitochondrial genome database (www.mitomap.org/MITOMAP) the alteration is only reported as a mtDNA coding region sequence variant and has not been reported in association with disease yet. Several simulations (prediction tools) assume a benign change (fathmm, MutationTaster, PROVEAN), according to SIFT it is a pathogenic variant (Table 1). Besides the mtDNA analysis, only the nuclear DNA polymerase subunit gamma (*POLG*) gene was analyzed in the patient. In the *POLG* gene (part of mitochondria-related nDNA) no pathogenic variant (mutation), but the well-known polymorphism c.156_158delGCA; p.Q55del (rs727504102) was detected in heterozygous state. For this deletion, an association with diabetes mellitus has been reported in the Human Gene Mutation Database (http://www.hgmd.cf.ac.uk/ac/index.php). It is classified as a clearly benign polymorphism in several databases (https://www.ncbi.nlm.nih.gov/clinvar/variation/167521/).

**Table 1:** Upper part: Different *in silico* prediction scores for the pathogenic relevance of the novel base exchange variant in the *MT-ND4* gene of our patient.

**Pathogenicity prediction tool**	**Transcript**	**Prediction**
FATHMM	ENST00000361381	Tolerated
MutationTaster	ENST00000361381	Polymorphism
Provean	ENST00000361381	Neutral
SIFT	ENST00000361381	Damaging
**mtDNA databases**	**Allele frequency**	**Prediction**
MITOMAP	2/46,092 mtDNA sequences	Coding region sequence variant
HmtVar	Healthy total: 0.000048	Likely polymorphic

Lower part: Information on the present sequence variant from two mitochondrial DNA (mtDNA) databases; MITOMAP: www.mitomap.org; HmtVar: www.hmtvar.uniba.it/varCard/22365

### Psychiatric Examination and Somatic Investigations

**Figure 3:**
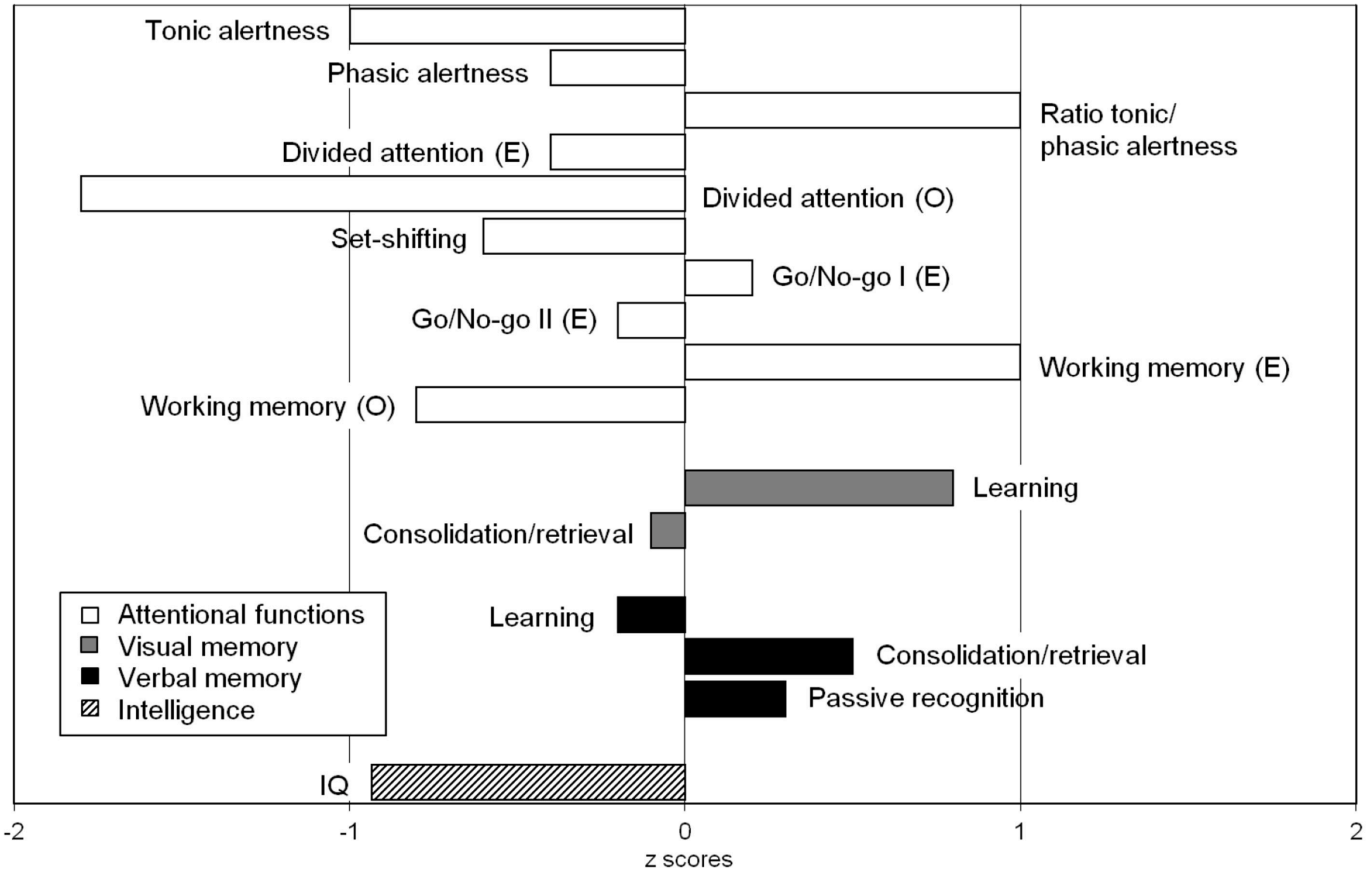
Neuropsychological findings showed significant impairments in divided attention for visual and auditory stimuli combined. The recorded intelligence level was in the lower normal range (IQ = 86), which appeared discrepant with the patient's educational background (this may also be attributed to fluctuations in attention). A detailed description of all test results can be found in Table 2 in the bottom column on neuropsychological test results.

The psychiatric investigation showed attention and concentration deficits, memory difficulties, affective instability, impulsive behaviors and sensory overload. Cognitive performance showed partially below-average results (Figure 3). The lactate ischemia test revealed a more than three-fold increase in lactate levels compared to resting state values. The lactate concentration in the cerebrospinal fluid (CSF) was not increased. In the muscle biopsy, single cytochrome c oxidase (COX)-negative muscle fibers were identified. In the immunological screening, increased anti-thyroglobulin, increased anti-thyroid peroxidase, anti-adrenal cortex, anti-glutamic acid decarboxylase (GAD-65), anti-partial cell, anti-intrinsic factor, and anti-nuclear antibodies (ANAs) were discerned. Serum concentration for immunoglobulin (Ig)G, IgM and especially IgA were within normal range. The B-cell panel showed clearly increased number of IgD-CD27+B-cells, IgA+CD27+memory B-cells, and IgM+/–CD38++plasma blasts of B-cells. The urine analyses revealed only slightly increased albumin-creatinine quotient and therefore no typical signs of focal segmental glomerulosclerosis (10). A current gastroscopy showed signs of autoimmune gastritis. The examination's findings are summarized in Table 2.

**Table 2:** Psychiatric examination, family history, somatic and neuropsychological findings.

Psychiatric examination	•Attention and concentration deficits, memory difficulties, affective instability, impulsive behaviors and sensory overload. •No depressiveness, no anxiety, no delusions/hallucinations, no suicidal tendencies, no sleep disorders, normal appetite.
Physical examination	•Internal: Normal internal investigation. •Dermatological: Diffuse hyperpigmentation, periorbital melasma, discreet acne (most likely steroid-triggered), xerosis skin, secondary hair was reduced. •Neurological: Monopod right somewhat plumped up, muscle self-reflexes at the upper and lower extremities right-emphasized, the remaining neurostatus was inconspicuous.
Family history	•Mother, sister, and maternal grandfather suffered from type 1 diabetes mellitus. Maternal grandfather died of kidney failure. •Several family members suffer from hearing impairments (paternal great-grandmother, paternal grandfather and grandmother, father; no investigations for underlying reason carried out). Paternal grandfather developed dementia in old age and presently suffers from bronchial carcinoma in his advanced age. •In terms of body height our patient was 172 cm tall but still the shortest in her family, her one sister is 174 cm (24 years), her other sister is 180 cm (22 years). Her parents are measuring both about 180 cm.
Genetic testing^*^	•Heteroplasmic base exchange mutation in the *MT-ND4* gene (m.12015T>C; p.Leu419Pro); MAF >0.01%; heteroplasmy level in the blood: 12%, in the muscle tissue 15%. •In the *POLG* gene, the well-known polymorphism c.156_158delGCA; p.Q55del (rs727504102) was found in heterozygous state.
Basic blood/urine analyses	•Blood cell count, electrolytes, liver/kidney/pancreas values, HbA1c, and folic acid were normal. Vitamin B12 was elevated under substitution (3,106 pg/ml; reference 197–771 pg/ml). Selenium was slightly reduced (68 μg/l; reference: 75–140 μg/l) •Thyroid-stimulating hormone and triiodothyronine levels were in normal ranges. Thyroxine was slightly increased under substitution: 22.2 pmol/l; reference: 12–22 pmol/l). •Normal blood glucose day profile. •Testosterone was not measurable (< 0.087 nmol/l). •ACTH in the morning (at 8 o'clock) was increased (243 pg/ml, reference: 10–60 pg/ml). •The urine analyses showed slightly increased albumin creatinine quotient (29 mg/g creatine). The urine-stix was slightly positive for protein (+) and negative for blood.
Immunological blood testing	•No antibodies against the intracellular onconeural antigens Yo, Hu, CV2/CRMP5, Ri, Ma1/2, SOX1 or the intracellular synaptic antigen amphiphysin were found. Anti-GAD65 antibodies were positive (quantification with ELISA-technique showed concentrations of 113 U/ml; reference < 2 U/ml). •Antibodies against neuronal cell surface antigens (*NMDAR, AMPA-R, GABA-B-R, VGKC-complex [LGI1, Caspr2]*) were negative. •Autoantibodies against thyroglobulin (329.3 IU/ml; reference < 115 IU/ml) and thyroid peroxidase (401 IU/ml; reference: < 34 IU/ml) were increased. Antibodies against the TSH receptor were normal. Adrenal cortex antibodies were increased (1:30; reference < 1:10). •Anti-partial cell and anti-intrinsic factor antibodies were positive. Anti-insulin and anti-IA2 antibodies were not increased. •Screening for antinuclear antibodies (ANA) in IIF showed a homogeneous nuclear fluorescence with a chromosomal pattern (1:200). Testing for extractable nuclear antibodies (ENA) showed no antibody specificity including anti-DFS-70 antibodies. Anti-neutrophil cytoplasmic antibodies, antiphospholipid antibodies, rheumatoid factor, and anti-mitochondrial antibodies were negative. •No changes in the complement system (C3, C4, CH50, C3d) were observed. •Serum immunoglobulin concentrations: 10.4 g/L IgG (reference 7.00–16.00 g/L), 1.05 g/L IgM (reference 0.7–4.0 g/L), 1.75 g/L IgA (reference 0.4–2.3 g/L). Immunofixation showed no monoclonal antibody production. •Lymphocyte and B-cell panel showed slightly increased levels of CD3+t-cells (84.1% reference: 55–83%), CD3+CD4+t-cells 58.2% (reference: 28–57%), clearly increased number of IgD-CD27+B-cells (29.88%, reference 5.7–24%), IgA+CD27+memory B-cells (14.72%, reference 2.8–10.9), and IgM+/-CD38++plasma blasts of B-cells (8.75%, reference 0.2–3.4%). CD65+CD16+natural killer cells (72/micro l, reference: 90–600/micro l) and CD56+CD16+natural killer cells (4.5%, reference: 7–31%) were reduced. All other cells were in the normal range.
Cerebrospinal fluid analyses	•Normal white blood cell count (1/μL; reference < 5/μL). •Normal protein concentration (247 mg/L; reference < 450 mg/L), and normal age-corrected albumin quotient: 2.5; age-dependent reference < 6.5 × 10^−3^). •No CSF specific oligoclonal bands; IgG index not increased (0.6; reference < 0.7). •CSF lactate normal (1.87 mmol/l; reference 1,5-2,1 mmol/L). •Antibodies against neuronal cell surface antigens (*NMDAR, AMPA-R, GABA-B-R, VGKC-complex [LGI1, Caspr2]*) were negative.
Cerebral magnetic resonance imaging (Figure 2)	•Substance defect of the parietooccipital left side exceeding the vascular territories with signs of chronic hemorrhagic transformation. •Small cortical/subcortical defects right temporal and frontoinsular. Cortical siderosis of a defect in the praecentral gyrus on the right and parietooccipital left.
Magnetic resonance spectroscopy^**^ (Figure 5)	•Single-voxel magnetic resonance spectroscopy results show slightly higher Lac+/Cr concentration ratio compared to control subjects: In the orbitofrontal cortex (OFC), a Lac+/Cr concentration ratio of 0.182 was found compared to a control group (males: N = 5, females: N = 1; 28 ± 2 years; Figure 5) with 0.159 ± 0.01, in the dorsal anterior cingulate cortex (dACC) 0.202 compared to 0.188 in one control subject (male, 31years) and in the dorsolateral prefrontal cortex (DLPFC) 0.172 compared to 0.168 in one control subject (male, 27years).
Electroencephalography	Alpha-rhythm, intermittent slowing accentuated left parieto-occipital; no epileptic patterns (under the treatment with high dose levetiracetam).
Electromyography	In the right anterior tibial muscle insertion activity was normal. No spontaneous activity. Evaluation of motor unit potentials revealed mild myopathic changes.
Lactate ischemia test	•Aerobic continuous load on the bicycle ergometer for 15 min at 30 watts: Resting lactate: 0.84 mmol/l, after 5 min: 2.35 mmol/l, after 10 min: 3.04 mmol/l, and after 15 min 3.25 mmol/l. •There was a continuous increase in heart rate over the course of 15 min.
Magnetic resonance imaging of thigh and lower leg	•Normal muscle signals.
Muscle biopsy	•Discrete substrate redistribution with partly sub-sarcolemmal, cap-like substrate propagation in the oxidative enzyme reactions, however, clear “ragged red/blue fibers” cannot be detected here. However, single COX-negative muscle fibers can be detected. •Ultrastructurally, small mitochondrial accumulations with individual, partly swollen, mitochondria can be found, but paracrystalline inclusions cannot be detected here. In addition, a discrete type 2 fiber atrophy can be detected.
Ophthalmological examinations	•Clinical ophthalmological examination was inconspicuous. •Macular scans were performed using spectralis optical coherence tomography device (spectral-domain OCT). Both eyes showed normal retinal volume and normal full retina thickness in all subfields with reference to normative data given by Nieves-Moreno et al. (11). •For visual electrodiagnostic testing light adapted electroretinogram was recorded from both eyes using RETeval device from LKC-Technologies. Both eyes showed normal cone function in response to the photopic-flash protocol and normal ganglion cell function with the stimulation protocol for the photopic negative response. Normative data were provided by LKC-Technologies (https://www.lkc.com/reteval-device-normative-data/).
Heart examinations	•Inconspicuous resting electrocardiography (ECG). The long-term ECG measurement showed a sinus rhythm with intermittent tachycardic pulse; no relevant pauses. •No ischemic changes and no load-inducible rhythm disturbances occur in the ECG during the lactatischemia test. •Transthoracic echocardiography was normal. •The external CT angiography of the thorax showed no detection of coronary anomalies^***^.
Otorhinolaryngologic examination	•Normal findings, no hearing impairment.
Ultrasound of the carotides	•Only increased flow velocities are noticeable in the arteria cerebri media and anterior cerebral artery on the right side^****^.
Thyroid sonography	•Echo-poor parenchyma and inhomogeneous perfusion. No noddings. Compatible with autoimmune thyroiditis^*****^.
Gastroscopy	•Mild chronic, inactive antral gastritis with foveolar hyperplasia. Clear chronic, moderately active corpus gastritis with intestinal metaplasia of the paneth- and bursa cell type.
Neuropsychological tests	•Test for attention performance: Borderline average/below average tonic alertness. Phasic alertness was found to be ordinary, the ability to increase response readiness was uncompromised. She exhibited an ordinary set-shifting ability/cognitive flexibility in the presence of an above average speed-accuracy trade-off index (T = 64) indicating a slowing in response times to increase response accuracy. She showed significant impairments in divided attention (visual and auditory stimuli combined) and working memory functions (2-back task). Performance was ordinary in both the simple (1 out of 2) and the complex (2 out of 5) go/no-go task. •Culture Fair Intelligence Test*-2*0*:* Results suggested an intelligence level in the lower normal range (IQ = 86) which appeared discrepant with the patient's educational background (may also be attributed to fluctuations in attention). •Route learning task: She was able to spontaneously recall the learned route correctly. She did not deviate from the route and therefore required no assistance by the experimenter. In the route recognition trial, she correctly identified the previously learned route as such. When asked to recall the designated landmarks, she spontaneously recalled eight out of the ten landmarks. In the cued condition, she recalled nine out of ten landmarks correctly. In the image-based recognition trial, she identified all landmarks as such. None of the distractor items were erroneously labelled as landmarks, resulting in a recognition accuracy of 100%. When tested 1 week later, she still recalled the route without error. •Verbal fluency*:* Was found to be below average in all subtests (words with S, category animals, alternating words with G-R, alternating categories sports-fruits). •Zoo map test*:* Revealed impairments in spontaneous planning ability. She was able to solve the given task correctly but only after amount of time (~8 min). When provided with a strategy (i.e., the correct sequence of stops on the map), she solved the task correctly in under 2 min. •Verbal learning and memory test*:* She showed ordinary verbal learning performance (*T* = 48) reproducing a total of 55 words (7-10-13-13-12) over the course of the five learning trials. Relative delayed recall performance (-2 words, *T* = 55) and passive recognition performance (*T* = 53) were ordinary. •Visual reproduction task: She exhibited quantitatively normal visual learning performance. Immediately after the learning trial, she recalled all geometric figures (38/41 points, *T* = 58). Delayed recall performance was similar (37/41 points, *T* = 49). Despite ordinary quantitative results, she made errors only with regard figure proportions but not figure components or their placement.

*The investigations were performed in August /September 2018 (a half year after the stroke like episodes and the Addison crisis)*.

* *The following regions were analyzed (in an external laboratory for human genetics): MT-ATP6, MT-ATP8, MT-CO1, MT-CO2, MTCO3, MT-CYB, MT-ND-1, MT-ND2, MT-ND3, MT-ND4, MT-ND4L, MT-ND5, MT_ND6, MT-RN1, MT-RNP2, MT-TA, MT-TC, MT-TD, MT-TE, MT-TK, MT-TL1, MT-TL2, MT-TM, MT-TN, MT-TP, MT-TQ, MT-TR, MT-TS1, MT-TS2, MT-TT, MT-TV, MT-TW, MT-TY (mitochondrial DNA) and POLG (mitochondria-related nuclear DNA)*.

** *Single-voxel magnetic resonance spectroscopy (MRS) examinations were performed in three prefrontal regions (orbitofrontal cortex [OFC], dorsal anterior cingulate cortex [dACC] and dorsolateral prefrontal cortex [DLPFC]) using a MEGA-sLASER difference editing sequence (12, 13), which is suitable for more robust lactate (Lac) quantification than is achievable with standard MRS protocols. Spectral quantification was performed with LCModel (14), using numerically simulated metabolite basis spectra, and Lac concentrations were reported as ratios to creatine (Cr). Since the Lac quantification results are slightly biased due to spectral overlap with coedited macromolecular resonances, the Lac concentration was denoted by Lac+ (15). The measured Lac+/Cr concentration ratios were compared with ratios obtained from healthy control subjects with the same protocol*.

*** *Already performed outside in October 2017*.

**** *Already performed outside in February 2018*.

***** *Already performed outside in March 2018*.

### Magnetic Resonance Imaging

**Figure 4:**
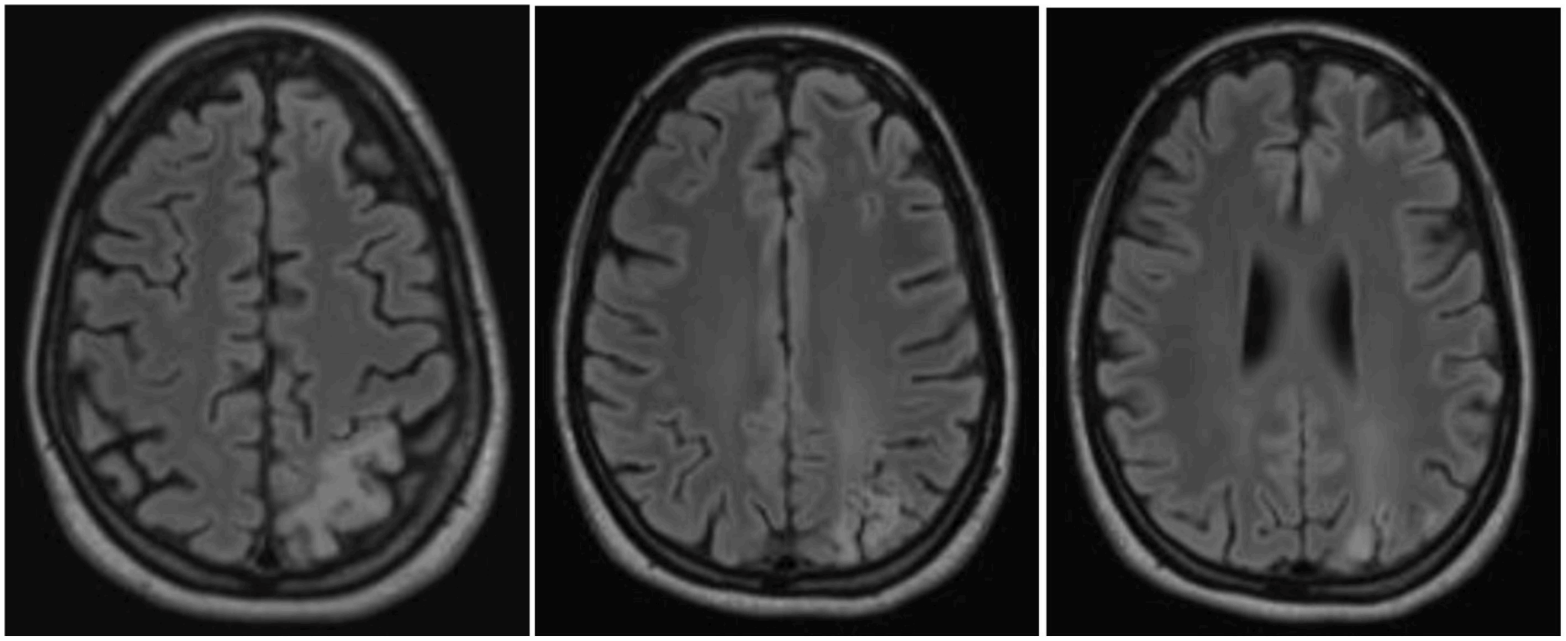
Magnetic resonance imaging findings a half year after the stroke-like episodes showed a chronic cortical to subcortical parieto-occipital brain lesion on the left side involving the posterior margin of the precentral gyrus with signs of chronic hemorrhagic transformation.

**Figure 5:**
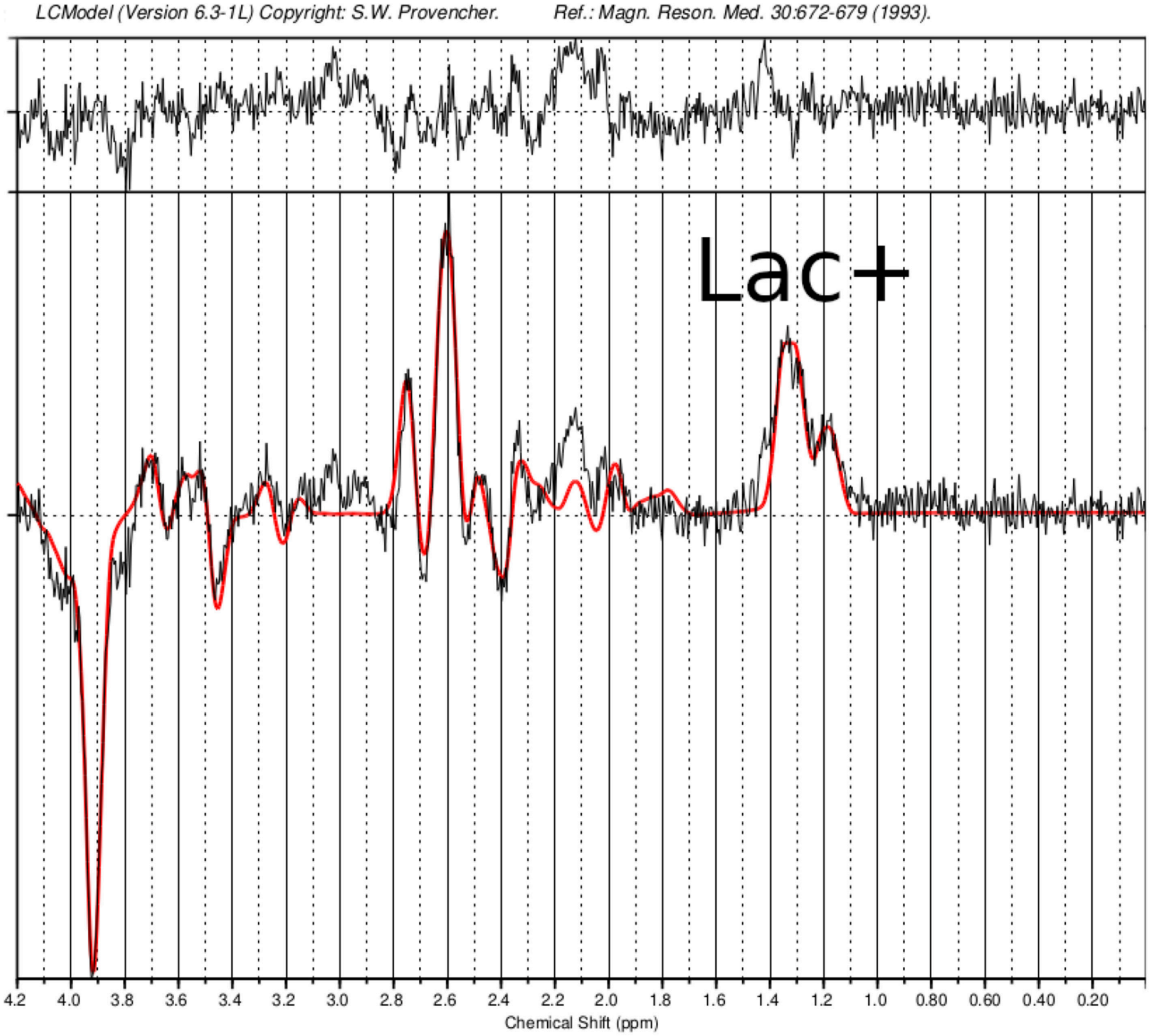
Single-voxel magnetic resonance spectroscopy imaging. Presented is a MEGA-sLASER difference spectrum from the orbitofrontal cortex (OFC) of the patient, with indicated lactate (Lac)+ resonance. In the OFC, a Lac+/Creatine concentration ratio of 0.182 was found compared to a control group (N=6; 5 males and 1 female; 28 ± 2 years) with 0.159 ± 0.01.

The follow-up cMRI 6 months after the stroke like events showed a chronic cortical to subcortical parieto-occipital brain lesion on the left side involving the posterior margin of the precentral gyrus with signs of chronic hemorrhagic transformation and the known multiple post-traumatic right hemispheric lesions (Figure 4). Single-voxel magnetic resonance spectroscopy results show slightly increased lactate (Lac)+/creatine ratios in the dorsal anterior cingulate cortex (dACC), dorsolateral prefrontal cortex (DLPFC), and orbitofrontal cortex (OFC; Figure 5).

## Discussion

In this paper, we present the case of a female patient with a new variant of MELAS with the clinical phenomenology of a dysexecutive syndrome and a history of two stroke-like episodes, “novel” heteroplasmic base exchange mutation in the *MT-ND4* gene (m.12015T>C; p.Leu419Pro), and comorbid polyglandular autoimmune syndrome type 2.

### Diagnostic Considerations

The patient suffered from myopathy with reduced physical capacity, a positive lactate ischemia test [following the criteria of Finsterer et al. (16)], discrete myopathic changes, and slightly pathological muscle biopsy results with single COX-negative muscle fibers and a discrete unspecific type 2 fiber atrophy. Myopathy clearly existed before steroid treatment began. Moreover, she suffered from encephalopathy with chronical dysexecutive syndrome, headache, EEG pathologies, and epileptic seizures (successfully treated with high doses of levetiracetam). She had two earlier stroke-like episodes with a typical parieto-occipital lesion and a lactate peak. Lactate levels outside the lesion (in the different prefrontal areas: ACC, OFC, and DLPFC) were slightly higher compared with controls, however the lactate concentration in the CSF was normal, which might indicate to a milder form of mitochondriopathy. Therefore, the patient fulfilled three main criteria of MELAS: Myopathy, encephalopathy and stroke-like episodes (3). The patient may have previously suffered from the fourth main symptom, lactate acidosis. Currently, the subject's serum and CSF lactate levels were normal. However, the result of the lactate ischemia test makes earlier episodes of lactate acidosis likely. With a body height of 172 cm, the patient does not have a short stature or growth hormone deficiency. However, she is the smallest person in her family. Her testosterone concentrations were extremely low (not measurable) and secondary hair was reduced. In a cohort of 99 MELAS patients abnormal growth was described in 50% of the individuals (8). No involvement of eyes, inner ears, the heart, or kidneys was found.

A diagnosis of Hashimoto's thyroiditis was made based on the clinical findings of hypothyroidism combined with increased antibodies against thyroglobulin and thyroid peroxidase, and with typical alterations in thyroid sonography. The findings to justify the diagnosis of Addison's disease were hyperpigmentation, loss of weight, gastrointestinal symptoms, and a history of Addison crisis with hypotension, diarrhea/vomiting, hypoglycemia, and vigilance reduction, alongside with metabolic acidosis and compatible laboratory findings (anti-adrenal cortex antibodies and increased ACTH-levels) and the normalization of these symptoms following treatment with gluco- and mineralocorticoids. There might be an overlap between Addison's disease and MELAS syndrome leading to the symptom of vomiting (could also be due to lactate acidosis in the context of MELAS). IgA serum concentration was within normal range and there was no evidence for mucocutaneous candidiasis, enamel hypoplasia, or hypoparathyroidism in the patients' medical history. Therefore, the diagnosis of APECED or autoimmune polyglandular syndrome type I (APS-I) was very unlikely. The combination of Hashimoto's thyroiditis, Addison's disease, and pernicious anemia led to the diagnosis of the more frequent autoimmune polyglandular syndrome type II. The course of MELAS syndrome was complicated by comorbid Addison's disease. Moreover, the present patient suffered from an autoimmune gastritis with anti-partial gastric cells, and further immunological activation with antibodies against GAD-65 (without clear signs for APECED), intrinsic factor, and nuclear antigens with negative DFS70 testing. Currently, the patient was not experiencing manifest type 1 diabetes. The finding of an ANA with homogeneous nuclear staining pattern in indirect immunofluorescence was suspicious for connective tissue disease like systemic lupus erythematosus. Because of the low ANA titer (1:200), no specificity in ENA-testing, unremarkable complement diagnostics, and no clinical symptoms indicative of a connective tissue disease (such as joint pain) this diagnosis was excluded.

### Genetic Aspects

**Figure 6:**
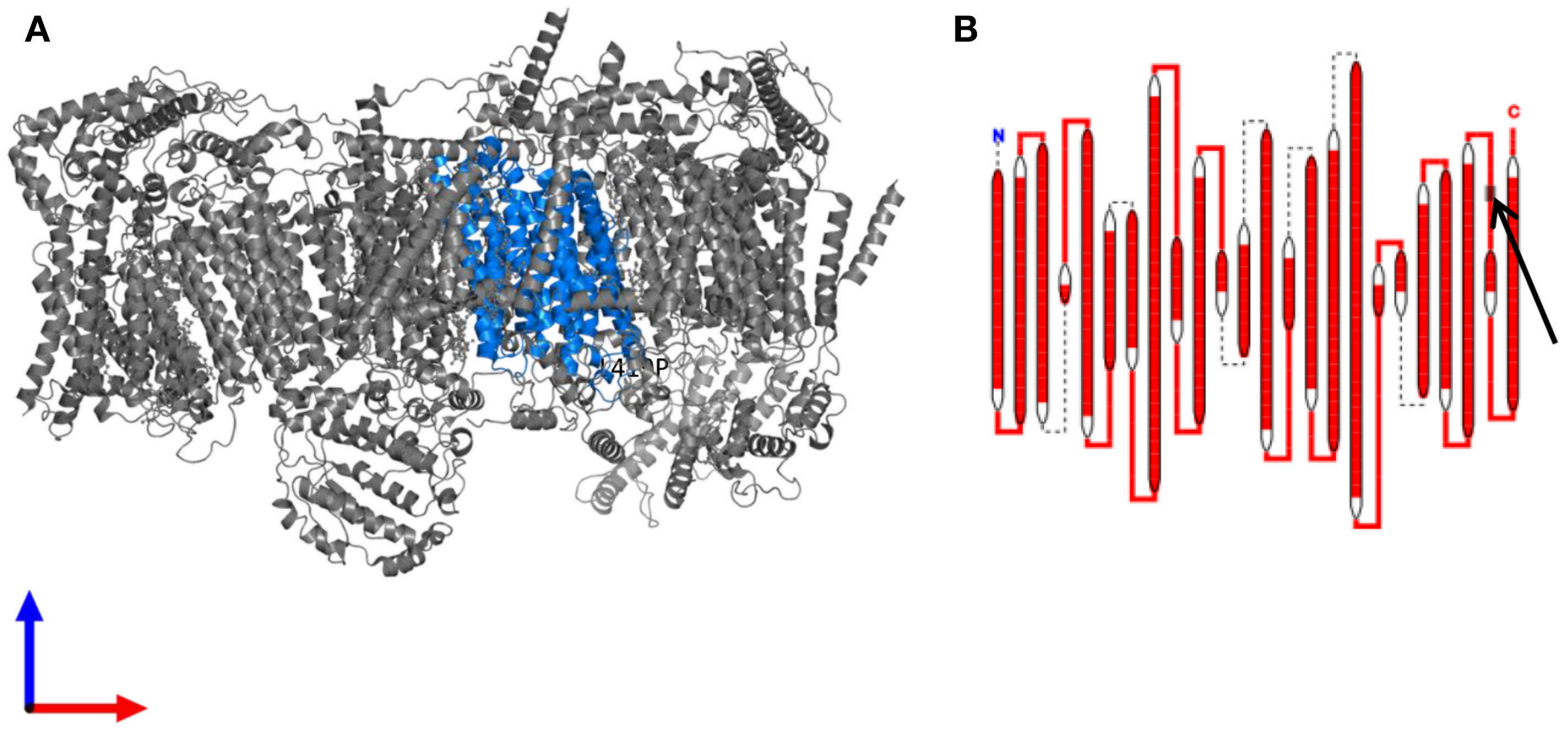
The resulting protein complex with the changed protein **(A)** Structure of the human respiratory chain complex I. The protein subunit carrying the mutation is highlighted in blue. **(B)** Schematic drawing of the complex I subunit altered in the patient. The site of the mutation in an extramembranous domain is indicated by an arrow. Data are derived from https://www.ebi.ac.uk/pdbe/entry/pdb/5xtc/protein/25 (20).

MELAS syndrome is classically (in over 80% of all cases) associated with a point mutation with adenine-to-guanine transition at position 3243 of mtDNA (m.3243A>G) in the mitochondrially encoded tRNA leucine 1 *(MT-TL1)* gene encoding tRNA^Leu(UUR)^ (1, 3). Further underlying mutations were found in the mitochondrial genes *MT-TL1* (in particular, mutation 3271T>C) (17), *MTTQ, MTTH, MTTK, MTTC, MTTS1, MTND1, MTND5, MTND6, and MTTS2*. One case report describes a case of MELAS syndrome due to mutation in the nuclear *POLG1* gene (18). The present patient displayed a heteroplasmic base exchange mutation in the *MT-ND4* gene (m.12015T>C; p.Leu419Pro). Changes in the *MT-ND4* gene were earlier published in one paper regarding two patients with MELAS syndrome (PubMed search for “MELAS” and “ND4” performed on December 23, 2018). An A-to-G base substitution at 11084, which leads to a Thr to Ala amino acid substitution was suggested to be disease-related (19). That association was later proven wrong as this very mutation was found in 14% of Asians (thus classifying it as a polymorphism). The point mutation affecting the patient described here has thus far only been detected in two of 46,092 examined sequences and has not yet been associated with mitochondriopathy (https://www.mitomap.org/MITOMAP and HmtVar: www.hmtvar.uniba.it/varCard/22365. It is possible that the two subjects with the same variant, who were documented earlier, had less-severe heteroplasmia, so that penetrance was low, i.e., the critical threshold value, which leads to the appearance of symptoms, was not exceeded. A very low and therefore undetectable heteroplasmia level in blood or a *de novo* mutation might explain the inconspicuous findings in the mtDNA of the present patients' mother. An examination of the asymptomatic mother from a second tissue was not desired. The resulting protein complex with the changed protein in the present patient (see Figure 6; marked in blue) is part of the respiratory complex I (https://www.ebi.ac.uk/pdbe/entry/pdb/5xtc/protein/25; Figure 6), which is comparable with genetic alterations in earlier published cases with MELAS syndrome (1). Different simulations resulted in contradictory results (from mostly benign to pathophysiologically relevant). From a clinical perspective, the association seems logical (the phenotype, especially including the results of the muscle biopsy and the MRI underline a clinical picture of MELAS), however, currently there is not enough evidence available to prove the association. Further data on patients with a similar phenotype harboring the same *MT-ND4* alteration are needed to evaluate the association of this novel mtDNA point mutation and MELAS syndrome. Moreover, there might be further mutations or variants in the nDNA regarding Addison's disease or polyglandular autoimmune syndrome, which could contribute to this complex disorder.

### Immunological Aspects

The most common immunological comorbidity in MELAS syndrome is type 1 diabetes mellitus (4), especially due to the frequent m.3243A > G mutation in MELAS syndrome, which is associated with diabetes mellitus (7). The present patient had increased diabetes-associated anti-GAD65 antibodies and a carried a polymorphism in the *POLG* gene, which is known to be associated with diabetes mellitus; however, clinically, she did not suffer from diabetes mellitus. The prevalence of growth and sexual hormone alterations was also high in earlier studies (8). In line with that observation, our patient suffered from low testosterone levels and is the shortest in her family. Hashimoto's thyroiditis and Addison's disease were earlier described as comorbid disorders in patients with mitochondriopathies; however, generally they do not seem to occur more often in this condition (7, 8). The association between MELAS syndrome and Addison's disease was explored in earlier papers (21, 22). O'Grady and colleagues presented an overview of earlier cases with a combination of mitochondriopathy and primary adrenal insufficiency. All the cases had manifested in childhood and lacked predominant psychiatric symptoms (i.e., dysexecutive symptoms), as was the case with our patient (22).

To our knowledge, this is the first case report of a patient with MELAS syndrome with comorbid autoimmune polyglandular syndrome type 2. This aspect is clinically important, as shown in the patient's history. Addison's disease and MELAS syndrome (lactate acidosis/myopathy) share clinical features with respect to weakness, fatigue, and abdominal complaints such as nausea or diarrhea. Hence, it is important to think of both diseases when considering the differential diagnosis. Additionally, our patient was tested positive for further antibodies. It is important to recognize these immunological changes to prevent end organ damage. In our patient, gastritis or anemia could be avoided by administering stomach protection and a vitamin B12 substitute, Addison's disease was successfully treated with steroids; L-thyroxin was substituted, and regular controls for early detection of diabetes mellitus were recommended.

Autoimmune polyendocrine syndromes are frequently associated with a broad spectrum of antibodies. In contrast, mitochondrial disorders like MELAS are generally not characterized by the presence of antibodies or autoimmune phenomena. Therefore, it is most likely that the broad spectrum of antibodies described here is part of the APS in our patient. On the other hand, it cannot be excluded for sure that mitochondrial disorders themselves may have impact on components of the immune system. Like neurons, cardiac myocytes, and skeletal muscle fibers, which are frequently affected in mitochondrial disorders because of their postmitotic state and longevity, antibody-producing plasma cells are postmitotic, long-living cells and highly dependent on sufficient energy supply by their mitochondria. A decline in ATP production could impair the quantity of ab production and result in hypogammaglobulinemia. An increased formation of reactive oxygen species might result in increased mutation rates and activation of apoptotic pathways within immune cells. Since mitochondria also play an important role in intracellular calcium homeostasis mitochondrial disorders might affect cellular mobility and signaling processes (8).

In summary, to our knowledge this is the first case presentation of a patient with atypical MELAS syndrome presenting with a neuropsychiatric syndrome of predominantly executive dysfunction, together with a polyglandular autoimmune syndrome type 2, and a heteroplasmic point mutation in the *MT-ND4* gene. The recognition of such constellations is important for further research and clinical differential diagnosis in complex neuropsychiatric cases.

## Data Availability

All datasets generated for this study are included in the manuscript and/or the supplementary files.

## Ethics Statement

The patient has given her signed written informed consent for this case report, including the presented images, to be published.

## Author Contributions

DoE treated the patient, performed the data research, and wrote the paper. PS, KK, KD, and LT supported the interpretation. SD, DaE, and PS performed the neuropathological analyses and interpretation. KE performed and interpreted the MRIs. SM, KN, MD, and TL performed and interpreted the magnetic resonance spectroscopy. FS, CZ, BF, and KK interpreted the genetic testing. FG performed the neurological investigation and interpretation. NV performed the rheumatological measurements and interpreted the results. EF performed the opthalmoscopic examinations. All authors were critically involved in the theoretical discussion and composition of the manuscript. All authors read and approved the final version of the manuscript.

### Conflict of Interest Statement

LT has received fees for lectures from Medice, Sevier, Shire, and Actelion within the last three years. The remaining authors declare that the research was conducted in the absence of any commercial or financial relationships that could be construed as a potential conflict of interest.
